# Editorial: Methods in Pediatric Critical Care 2022

**DOI:** 10.3389/fped.2023.1158611

**Published:** 2023-03-09

**Authors:** Artur F. Delgado

**Affiliations:** Hospital das Clínicas, São Paulo University, São Paulo, Brazil

**Keywords:** children, sepsis, nutritional, calorimetry, nutrition, arificial intelligence

**Editorial on the Research Topic** Methods in Pediatric Critical Care 2022

It is very difficult to have homogeneous population to study determined clinical conditions and interventions, mainly in children and adolescents with sepsis/septic shock with nutritional impairment. Sepsis can deteriorate nutritional status in a short period of time in hospitalized patients contributing to increased morbidity, mortality, and costs, especially in the critically ill population (1).

Early identification of severe sepsis through a combination of danger signs of end-organ dysfunction or impaired circulation is vital to improve outcome. Fluid therapy with crystalloids carries on a cornerstone in the treatment of septic patients. The risk of overhydration in malnourished children leading to interstitial, pulmonary, and cerebral edema has resulted in recommendations of cautious fluid administration in modest amounts. For children with severe acute malnutrition without signs of severe shock, a careful administration of intravenous fluids at an initial rate of 10–15 ml/kg/h (no fluid boluses) should be used (2–4). In this edition of Methods in Pediatric Critical Care 2022, Yenjabog et al. emphasize the importance to avoid fluid overload that increases mortality. The authors show a critical appraisal of more advanced approach of hemodynamic monitoring in predicting fluid responsiveness in mechanically ventilated children using the respiratory variation in aortic peak velocity in perioperative (including congenital heart surgery) and clinical conditions (5). Lee et al. (6). in a review article about hemodynamic monitoring and management of pediatric septic shock cited two systemic reviews that demonstrated respiratory variation in aortic blood flow peak velocity, as measured by ultrasound, can be an accurate predictor of fluid responsiveness in ventilated children (level of evidence: 1B) (7, 8).

Systemic Inflammatory Response Syndrome (SIRS) can progress to septic shock if not identified early. There are several clinical and laboratorial parameters used to identify aspects related to the evolution of these conditions. The predictors of mortality most found are: late hospital admissions, abnormal leukocyte count, positive blood cultures, and severe acute malnutrition (9). The identification, classification and adequate monitoring of nutritional status is essential and differences occur among patients with similar genetics and environmental influences. In a case-control study nested within a multicenter randomized controlled trial among children with a complicated severe malnutrition in developing countries, the authors found that blood metabolomic and proteomic profiles robustly differentiated children who died (*n* = 92) from those who survived (*n* = 92) and these data reinforce other recent study with similar outcome (10, 11). In this edition of Methods in Pediatric Critical Care 2022, Silva-Gburek et al. discuss about a clinical and methodological approach to measure energy expenditure in the critically ill pediatric patient reviewing the utilization of indirect calorimetry, considering the gold standard method, to evaluate energy expenditure and substrate utilization by measuring gas exchange in exhaled air and urinary nitrogen (12). Regarding to cost-benefit ratio, this evaluation can measure energy requirements in a precise way and optimize nutritional therapy avoiding degradation of nutritional status and worsening prognosis. Clinical trials in sepsis have mainly been focused on targeting the inflammatory pathway however metabolic dysregulation takes place in sepsis, and metabolic outcome might hold much promise for the management of sepsis (13). Experimental data indicate that sepsis influences the mitochondrial function and metabolism. A pattern of early longitudinal induction of metabolic-hormones, repression of bioenergetics and innate immunity, hypo-metabolism, and amino-acid kinetics changes discriminate sepsis from SIRS; malnutrition, hypo-metabolism, and persistently increased resistin and adiponectin are associated with poor outcome (14).

Predicting mortality in septic children and adolescents, including those with undernutrition, remains a challenge. The use of scoring systems to predict mortality, mainly in under-five children with frequent nutritional status involvement, can be a determinant factor that could be considered, mainly in emergent countries (15, 16). In this edition of Methods in Pediatric Critical Care 2022, Recher et al. performed an updated state-of-the-art about the main severity and organ dysfunction scoring systems in pediatric intensive care (17). These scores can help clinicians understand much better about monitoring and therapeutics attitudes in PICU.

Scoring systems and other databases can represent critical elements of the course of action that is applied in order to intervene in the reality of facts. Considering that heterogenous patient populations in pediatric critical care with potential small case numbers is frequent and constitute a relevant barrier to research, population-based administrative health care data will remain a major source to evaluate the epidemiology of diseases and diagnostic or therapeutic strategies that lack evidence (18). Codes for “severe sepsis” and “septic shock” can identify smaller but higher acuity cohorts of patients that more closely resemble the children enrolled in the largest clinical trials of pediatric severe sepsis to date (19). In this edition of Methods in Pediatric Critical Care 2022, Bruns et al. discuss challenges to the use of administrative data by clinicians highlighting that retrospective observational investigations, mainly multicenter studies or analyses of registry data, prevail in the field of pediatric critical care research but facing many obstacles (20, 21).

Future directions that may lead to a more precision-based approach to sepsis recognition and treatment are necessary. Artificial intelligence can be used to predict the onset of severe sepsis using physiomarkers in critically ill children. Further, it may detect severe sepsis as early as 8 h prior to a real-time electronic severe sepsis screening algorithm (22, 23). Clinical data and some biomarkers can be used in a systematic form to create real phenotypes with potential different evolution and prognosis. The characterization of different disease patterns can facilitate the establishment of early monitoring and therapeutic measures. Children and adolescents with malnutrition and sepsis can have a pattern of clinical and laboratorial similarities that can characterize a model of prognostic evolution when using artificial intelligence, facilitating future research (24). Park et al. in this edition of Methods in Pediatric Critical Care 2022, emphasize that is critical to avoid “one-size-fits-all” approaches and to employ a precision medicine is useful, remind us to accelerate the early diagnosis and treatment of pediatric sepsis. Several phenotypes have been identified based on prognostic biomarkers that are empirically selected. In a polished discussion they approached: sample size examination, missing data handling, correlation adjustment, cluster number determination and phenotype validation (25).

We reckon that it is still very difficult to diagnose and treat pediatric patients with sepsis/septic shock mainly if they are severely undernourished living in a developing country and refine subgroups can help clinicians to diagnose and establish early treatment with a consequent better prognosis. More efforts and investments are needed for the creation of these models and adequate use of new technologies in future research.

## References

[1] McLaughlin J Chowdhury N Djurkovic S Shahab O Sayiner M Fang Y Clinical outcomes and financial impacts of malnutrition in sepsis. Nutr Health. (2020) 26(3):175–8. 10.1177/0260106020930145 32571151

[2] World Health Organization. Guideline: Updates on the management of severe acute malnutrition in infants and children. Geneva: World Health Organization (2013). 24649519

[3] Maitland K Kiguli S Opoka RO Engoru C Olupot-Olupot P Akech SO Mortality after fluid bolus in African children with severe infection. N Engl J Med. (2011) 364:2483–95. 10.1056/NEJMoa1101549 21615299

[4] Ribeiro CT Delgado AF de Carvalho WB. Mortality after fluid bolus in African children with sepsis. N Engl J Med. (2011) 365(14):1348–9. 10.1056/NEJMc1108712 21991961

[5] Yenjabog P Kanchongkittiphon W Chutipongtanate S Lertbunrian R Ungprasert P. Dynamic parameters for fluid responsiveness in mechanically ventilated children: a systematic review. Front Pediatr. (2022) 10:1010600. 10.3389/fped.2022.1010600 36353262 PMC9638161

[6] Lee EP Wu HP Chan OW Lin JJ Hsia SH. Hemodynamic monitoring and management of pediatric septic shock. Biomed J. (2022) 45(1):63–73. 10.1016/j.bj.2021.10.004 34653683 PMC9133259

[7] Gan H Cannesson M Chandler JR Ansermino JM. Predicting fluid responsiveness in children: a systematic review. Anesth Analg. (2013) 117:1380–92. 10.1213/ANE.0b013e3182a9557e 24257389

[8] Desgranges FP Desebbe O Pereira de Souza Neto E Raphael D Chassard D. Respiratory variation in aortic blood flow peak velocity to predict fluid responsiveness in mechanically ventilated children: a systematic review and meta-analysis. Paediatr Anaesth. (2016) 26:37–47. 10.1111/pan.12803 26545173

[9] Shah S Deshmukh CT Tullu MS. The predictors of outcome and progression of pediatric sepsis and septic shock: a prospective observational study from western India. J Postgrad Med. (2020) 66(2):67–72. 10.4103/jpgm.JPGM_171_19 31997781 PMC7239413

[10] Wen B Njunge JM Bourdon C Gonzales GB Gichuki BM Lee D Systemic inflammation and metabolic disturbances underlie inpatient mortality among ill children with severe malnutrition. Sci Adv. (2022) 8(7):eabj6779. 10.1126/sciadv.abj6779 35171682 PMC8849276

[11] Chowdhury VP Sarmin M Kamal M Islam S Siddik MA Afroze F Factors associated with mortality in severely malnourished hospitalized children who developed septic shock. J Infect Dev Ctries. (2022) 16(2):339–45. 10.3855/jidc.15135 35298430

[12] Silva-Gburek J Zhu PH Mansour M Walding D Coss-Bu JA. A methodological and clinical approach to measured energy expenditure in the critically ill pediatric patient. Front Pediatr. (2022) 10:1027358. 10.3389/fped.2022.1027358 36353257 PMC9638495

[13] Vandewalle J Libert C. Sepsis: a failing starvation response. Trends Endocrinol Metab. (2022) 33(4):292–304. 10.1016/j.tem.2022.01.006 35181202

[14] Spanaki AM Tavladaki T Dimitriou H Kozlov AV Duvigneau JC Meleti E Longitudinal profiles of metabolism and bioenergetics associated with innate immune hormonal inflammatory responses and amino-acid kinetics in severe sepsis and systemic inflammatory response syndrome in children. J Parenter Enteral Nutr. (2018) 42(6):1061–74. 10.1002/jpen.1050 29338093

[15] Kapoor A Awasthi S Kumar Yadav K. Predicting mortality and use of RISC scoring system in hospitalized under-five children due to WHO defined severe community acquired pneumonia. J Trop Pediatr. (2022) 68(4):fmac050. 10.1093/tropej/fmac050 35727140

[16] Singh M Sankar J Kumar A Kumar UV Lodha R Kabra SK. Predictors of mortality in children admitted to the pediatric intensive care unit with acute gastroenteritis with severe dehydration. Indian J Pediatr. (2019) 86(12):1142–5. 10.1007/s12098-019-03094-0 31701427

[17] Recher M Leteurtre S Canon V Baudelet JB Lockhart M Hubert H. Severity of illness and organ dysfunction scoring systems in pediatric critical care: the impacts on clinician's practices and the future. Front Pediatr. (2022) 10:1054452. 10.3389/fped.2022.1054452 36483470 PMC9723400

[18] Marraro GA Spada C Zeng Y. Administrative database and empiric therapy are always useful for appropriate treatment of pediatric patients? Crit Care Med. (2020) 48(3):438–40. 10.1097/CCM.0000000000004175 32058384

[19] Hartman ME Saeed MJ Powell KN Olsen MA. The comparative epidemiology of pediatric severe sepsis. J Intensive Care Med. (2019) 34(6):472–9. 10.1177/0885066617735783 29034782 PMC6050143

[20] Bruns N Sorg AL Felderhoff-Müser U Dohna-Schwake C Stang A. Administrative data in pediatric critical care research-potential, challenges, and future directions. Front Pediatr. (2022) 10:1014094. 10.3389/fped.2022.1014094 36245724 PMC9554413

[21] Ames SG Davis BS Angus DC Carcillo JA Kahn JM. Hospital variation in risk-adjusted pediatric sepsis mortality. Pediatr Crit Care Med. (2018) 19(5):390–6. 10.1097/PCC.0000000000001502 29461429 PMC5935525

[22] Eisenberg MA Balamuth F. Pediatric sepsis screening in US hospitals. Pediatr Res. (2022) 91(2):351–8. 10.1038/s41390-021-01708-y 34417563 PMC8378117

[23] Kamaleswaran R Akbilgic O Hallman MA West AN Davis RL Shah SH. Applying artificial intelligence to identify physiomarkers predicting severe sepsis in the PICU. Pediatr Crit Care Med. (2018) 19(10):e495–503. 10.1097/PCC.0000000000001666 30052552

[24] Agor J Özaltın OY Ivy JS Capan M Arnold R Romero S. The value of missing information in severity of illness score development. J Biomed Inform. (2019) 97:103255. 10.1016/j.jbi.2019.103255 31349049

[25] Qin Y Bohn RC Sriram A Kernan K Carcillo JA Kim S Refining empiric subgroups of pediatric sepsis using machine-learning techniques on observational data. Front Pediatr. 11:1035576. 10.3389/fped.2023.1035576 36793336 PMC9923004

